# Common variants in glyoxalase I do not increase chronic pancreatitis risk

**DOI:** 10.1371/journal.pone.0222927

**Published:** 2019-10-29

**Authors:** Tom Kaune, Marcus Hollenbach, Bettina Keil, Jian-Min Chen, Emmanuelle Masson, Carla Becker, Marko Damm, Claudia Ruffert, Robert Grützmann, Albrecht Hoffmeister, Rene H. M. te Morsche, Giulia Martina Cavestro, Raffaella Alessia Zuppardo, Adrian Saftoiu, Ewa Malecka-Panas, Stanislaw Głuszek, Peter Bugert, Markus M. Lerch, Frank Ulrich Weiss, Wen-Bin Zou, Zhuan Liao, Peter Hegyi, Joost PH Drenth, Jan Riedel, Claude Férec, Markus Scholz, Holger Kirsten, Andrea Tóth, Maren Ewers, Heiko Witt, Heidi Griesmann, Patrick Michl, Jonas Rosendahl

**Affiliations:** 1 Department of Internal Medicine I, Martin Luther University, Halle, Germany; 2 Medical Department II–Gastroenterology, Hepatology, Infectious Diseases, Pulmonology, University of Leipzig Medical Center, Leipzig, Germany; 3 Institut National de la Santé et de la Recherche Médicale (INSERM), U1078, Etablissement Français du Sang (EFS)–Bretagne, Faculté de Médecine et des Sciences de la Santé, Université de Bretagne Occidentale, Brest, France; 4 Laboratoire de Génétique Moléculaire et d'Histocompatibilité, Centre Hospitalier Régional Universitaire (CHRU) Brest, Hôpital Morvan, Brest, France; 5 Universitätsklinikum Erlangen, Friedrich-Alexander-Universität Erlangen-Nürnberg, Chirurgische Klinik, Erlangen, Germany; 6 Department of Gastroenterology and Hepatology, Radboud umc, Nijmegen, The Netherlands; 7 Gastroenterology and Gastrointestinal Endoscopy Unit, Division of Experimental Oncology, Vita-Salute San Raffaele University, IRCCS Ospedale San Raffaele Scientific Institute, Milan, Italy; 8 Department of Internal Medicine and Gastroenterology, University of Medicine and Pharmacy, Craiova, Romania; 9 Department of Digestive Tract Diseases, Medical University of Łódź, Łódź, Poland; 10 Faculty of Medicine and Health Sciences, Jan Kochanowski University, Kielce, Poland; 11 Institute of Transfusion Medicine and Immunology, Medical Faculty Mannheim, Heidelberg University, German Red Cross Blood Service of Baden-Württemberg, Mannheim, Germany; 12 Department of Medicine A, University Medicine Greifswald, Greifswald, Germany; 13 Department of Gastroenterology, Changhai Hospital, Second Military Medical University, Shanghai Institute of Pancreatic Diseases, Shanghai, China; 14 Institute for Translational Medicine and First Department of Internal Medicine, Medical School, University of Pécs, Pécs, Hungary; 15 HAS-SZTE, Momentum Gastroenterology Multidisciplinary Research Group, Szeged, Hungary; 16 Institute for Medical Informatics, Statistics and Epidemiology, University of Leipzig, Leipzig, Germany; 17 LIFE- Leipzig Research Center for Civilization Diseases, University of Leipzig, Leipzig, Germany; 18 Else Kröner-Fresenius-Zentrum für Ernährungsmedizin (EKFZ), Paediatric Nutritional Medicine, Technische Universität München (TUM), Freising, Germany; Medizinische Fakultat der RWTH Aachen, GERMANY

## Abstract

**Introduction:**

Chronic pancreatitis (CP) may be caused by oxidative stress. An important source of reactive oxygen species (ROS) is the methylglyoxal-derived formation of advanced glycation endproducts (AGE). Methylglyoxal is detoxified by Glyoxalase I (GLO1). A reduction in GLO1 activity results in increased ROS. Single nucleotide polymorphisms (SNPs) of *GLO1* have been linked to various inflammatory diseases. Here, we analyzed whether common *GLO1* variants are associated with alcoholic (ACP) and non-alcoholic CP (NACP).

**Methods:**

Using melting curve analysis, we genotyped a screening cohort of 223 ACP, 218 NACP patients, and 328 controls for 11 tagging SNPs defined by the SNPinfo LD TAG SNP Selection tool and the functionally relevant variant *rs4746*. For selected variants the cohorts were extended to up to 1,441 patient samples.

**Results:**

In the ACP cohort, comparison of genotypes for *rs1937780* between patients and controls displayed an ambiguous result in the screening cohort (p = 0.08). However, in the extended cohort of 1,441 patients no statistically significant association was found for the comparison of genotypes (p = 0.11), nor in logistic regression analysis (p = 0.214, OR 1.072, 95% CI 0.961–1.196). In the NACP screening cohort SNPs *rs937662*, *rs1699012*, and *rs4746* displayed an ambiguous result when patients were compared to controls in the recessive or dominant model (p = 0.08, 0.08, and 0.07, respectively). Again, these associations were not confirmed in the extended cohorts (*rs937662*, dominant model: p = 0.07, logistic regression: p = 0.07, OR 1.207, 95% CI 0.985–1.480) or in the replication cohorts for *rs4746* (Germany, p = 0.42, OR 1.080, 95% CI 0.673–1.124; France, p = 0.19, OR 0.90, 95% CI 0.76–1.06; China, p = 0.24, OR 1.18, 95% CI 0.90–1.54) and *rs1699012* (Germany, Munich; p = 0.279, OR 0.903, 95% CI 0.750–1.087).

**Conclusions:**

Common *GLO1* variants do not increase chronic pancreatitis risk.

## Introduction

Chronic pancreatitis (CP) is a recurring inflammation of the pancreas with progressive fibrosis by tissue destruction that in some patients results in exocrine and endocrine pancreatic insufficiency [1]. Several studies have identified that the underlying pathomechanisms can range from premature intrapancreatic activation of proteases to local and systemic inflammatory processes, which are relevant for the initiation and progression of the disease [2]. Recently, it was demonstrated that oxidative stress (ROS) is involved in these inflammatory and fibrotic processes [3–5]. As advanced-glycation-end products (AGE) impact on ROS, they may contribute to CP development [6] as it was shown in acute pancreatitis [7].

“Dicarbonyl stress” indicates a cellular condition where α-oxoaldehyde metabolites accumulate, leading to an increased modification of protein and DNA which contribute to cellular dysfunction in ageing and disease. “Dicarbonyl stress” is mainly caused by methylglyoxal (MGO) that is formed as a by-product in glycolysis [8], ketone body metabolism and threonine catabolism [9–11]. MGO is highly reactive with nucleotides, phospholipids and proteins [12,13] with the result of a rapid formation of AGE. In addition, reducing sugars like glucose react with amino groups and trigger MGO formation and AGE generation in a non-enzymatic protein glycation within the Maillard reaction [6]. AGE themselves induce several detrimental processes on a cellular level and furthermore activate different signaling pathways via the RAGE receptor. Moreover, they induce ROS and have been associated with various disease entities [14].

For protection on the cellular level MGO is detoxified by the Glyoxalase system. Glyoxalase I (GLO1) catalyzes the conversion of α-oxo-aldehydes such as MGO and L-glutathione (GSH) to form the corresponding hemithioacethal S-D-lactoylglutathione [15]. In the next step, hydroxyacyl glutathione hydrolase (GLO2) converts S-D-Lactoylglutathione to D-lactate and GSH. Herein, GLO1 is the rate-limiting enzyme in this series of reactions [16].

Thus far, *GLO1* single nucleotide polymorphisms (SNPs) were associated with distinct inflammatory diseases. The *rs4746* (p.Ala111Glu) variant displayed a decrease of GLO1 enzymatic activity for the *A*-allele in lymphoblastoid cells of the brain [17,18]. In addition, *rs4746* has been linked to diabetes [19], atherosclerosis [20], chronic renal failure [21], vascular diseases [22,23], neuropsychiatric disorders [24,25], and different cancer types [26–29]. Moreover, *rs1130534* (c.372A>T, p.Gly124 = ) and *rs1049346* were correlated with lower enzyme activity, but did not associate with vascular complications in diabetes mellitus [30].

In conclusion, *GLO1* variants with a diminished GLO1 activity cause increased MGO levels and consecutive ROS generation. Therefore, we reasoned that *GLO1* SNPs contribute to the development of CP and investigated whether genetic variants in *GLO1* are associated with alcoholic CP (ACP) or non-alcoholic CP (NACP).

## Material and methods

### Patients and controls

The study was approved by the medical ethical review committee of the Martin-Luther-University of Halle-Wittenberg (Medical ethical committee, University Halle-Wittenberg, Medical Faculty, Bearbeitungsnummer 2015–106, date: 22.01.2016, title: “Erforschung molekulargenetischer Ursachen von Pankreaserkrankungen”). All patients gave written informed consent. The diagnosis of CP was based on two or more of the following findings: history of recurrent acute pancreatitis or recurrent or persisting abdominal pain typical for CP, pancreatic calcifications and/or pancreatic ductal irregularities indicated by computed tomography imaging, magnetic resonance imaging, endoscopic retrograde pancreaticography or (endo)sonography of the pancreas and/or the diagnosis of exocrine pancreatic insufficiency [31].

ACP was diagnosed in patients with a history of chronic alcohol intake (> 80 g per day for males or >60 g per day for females) for more than 2 years. NACP was diagnosed in the absence of known precipitating factors as alcohol consumption and/or smoking. Patients with a positive family history were included in the NACP group. The data on past ethanol consumption and the clinical presentation were based on research records and/or physician's history and/or completion of a detailed questionnaire by the patient.

Patients and Controls were recruited throughout Germany and in the European centres in The Netherlands, Romania, Poland, Italy, and Hungary. Controls were blood donors and healthy volunteers as described in our former publications [32].

**Fig 1:**
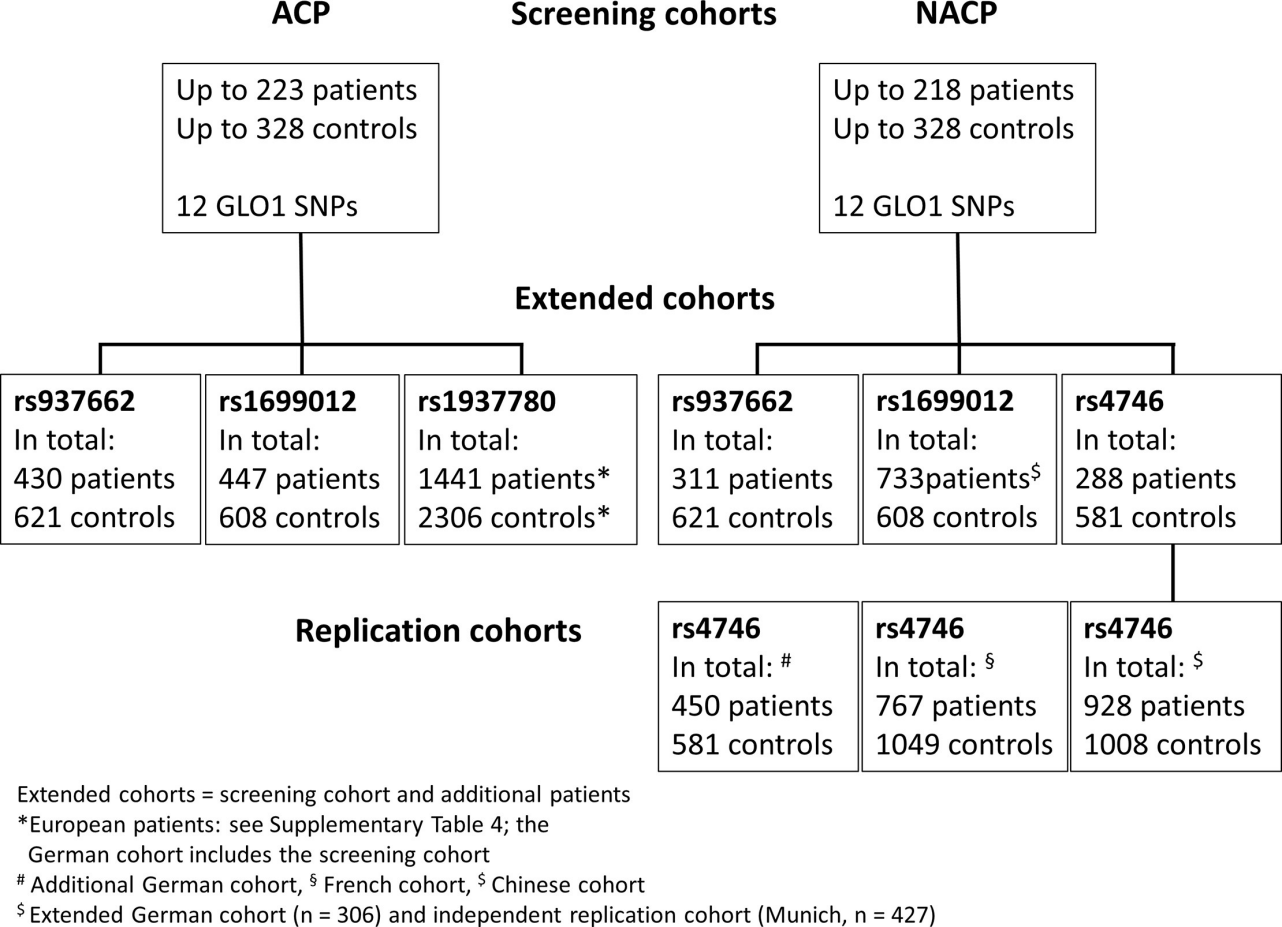
Flowchart of patients analyzed in this study. Note that the extended cohorts comprise the screening cohort and novel patients for all German patients (SNPs *rs937662*, *rs1937780*, and *rs4746*). For *rs1699012* the numbers summarize the extended with the replication cohort from Munich. The European cohorts (*rs1937780*) and the German, French, and Chinese replication cohorts for *rs4746* are additional patients.

In the screening cohort we investigated 223 ACP, 218 NACP patients, and 328 controls for 12 common *GLO1* SNPs with a minor allele frequency of a least 5%. Variants with nominal significance according to uncorrected p-values (*rs1699012*, *rs937662*, *rs4746*, and *rs1937780*) were analyzed in further subjects (extended cohorts). In addition, we screened European ACP cohorts for *rs1937780* and an additional German, French, and Chinese NACP cohort for *rs4746* and an independent German NACP cohort for *rs1699012* (see flow chart in **Fig 1**). For a detailed description of the screening cohort and the extended cohorts see **Table 1**.

**Table 1:** Description of the cohorts included in the analysis.

Cohort type	No.	Age (mean)	Age (median)	Age range	Male sex
Screening cohort (11 tagging SNPs + rs4746)					
Controls	328	63.9	63	60–70	49.4%
ACP	223	49.9	50	21–79	88.8%
NACP	218	39.6	39	5–80	56.2%
Screening cohort extended with additional German patients (rs1699012 and rs937662) and replication cohort from Germany (rs1699012)					
Controls	625	60.8	63	20–70	48.8%
ACP	451	49.9	50	21–79	82.3%
NACP	314	36.6	35	3–80	53.2%
NACP (Munich) *	427	19.2	14	0–71	51.1%
Screening cohort extended with additional German patients (rs4746)					
Controls	581	63.8	63	60–70	49.9%
ACP (German)	230	46.4	46	21–79	89%
NACP (German)	288	37.5	38	3–80	56.2%
Replication cohorts Germany and France (rs4746)					
Controls (German)	581	63.8	63	60–70	49.9%
NACP (German)	450	16.9	13	0–61	46.4%
Controls (French)	1049	45.7	40	24–63	56.2%
NACP (French)	767	39.7	35	4–93	56.0%
Controls (China)	504	41.0	41	18–62	66.9%
NACP (China)	464	45.0	46	5–91	71.6%
Screening cohort extended with additional German patients (rs1937780)					
Controls	1474	46.1	47	18–70	49.2%
ACP	872	44.7	45	14–85	87.3%
NACP	285	48.8	59	3–80	57.6%
Screening cohort extended with additional European and German patients (rs1937780)					
Controls	2306	47.8	48	18–99	48.1%
ACP	1441	44.7	45	14–98	85.0%

Note: The extended cohorts comprise the initial screening cohort and additional patients.

*Independent German replication cohort from Munich for *rs1699012*. Abbreviations: No., number of individuals; ACP, alcoholic chronic pancreatitis; NACP, non-alcoholic chronic pancreatitis.

### Selection of tagging SNPs in the *GLO1* locus

We selected 11 tagging SNPs in the *GLO1* locus using the SNPinfo LD TAG SNP Selection tool (LD map **Figure A in S1 File**). For this purpose we used an LD threshold of 0.8, a minimum of one SNP tagged, a minimum of 5 valid genotypes to calculate LD in populations with European ancestry (CEU) and extended the region of interest by 10.000 bp in the 5’-region and the 3’-region. In addition, we analyzed SNP *rs4746* that was described to be functionally relevant [17,18]. The other functionally relevant SNPs *rs1049346* and *rs1130534* were tagged by *rs1621788* and *rs13212218*, respectively. Details on the selected SNPs are summarized in **Table A in S1 File**.

### DNA extraction and SNP genotyping

DNA was isolated from EDTA blood using a commercial system (QIAamp Blood DNA Mini Kit; Qiagen, Hilden, Germany). Polymerase chain reaction (PCR) was conducted using OneTaq® 2X Master Mix (NEB) with 200 μM dNTPs, 1.8 mM MgCl_2_ and 0.1 μM forward primer as well as 0.1 μM reverse primer (0.4 μM forward primer for *rs3778443* and *rs17544798*; 0.2 μM reverse primer for *rs4746*) in a total volume of 25 μl. Cycle conditions were an initial denaturation at 95°C for 5 minutes followed by 45 cycles of 20 seconds denaturation at 95°C, 40 seconds annealing (**Table B in S1 File**), 90 seconds primer extension at 72°C followed by final extension for 5 minutes at 72°C in an automated thermal cycler. Primers and probes (**Table B in S1 File**) were synthesized by TIB Molbiol (Berlin, Germany). Genotyping was performed using the LightCycler480® system (Roche Diagnostics).

Probe oligomers were diluted in H_2_O to a concentration of 200 nM. For genotyping we used the PCR products from standard PCR (see above) with 50 nM (final) of probe oligomers followed by melting curve analysis with the following protocol: 95°C for 60 seconds, 40°C for 60 seconds, continuous increase to 70°C with various ramp rates (0.29°C/s *rs12198212* and *rs1621788*, 0.14°C/s *rs17544798*, *rs132212218* and *rs937662*, 0.19°C/s for the other seven SNPs). Call rates for all SNPs were > 95%. For quality control 2.6% of all samples were genotyped in duplicates blinded to the investigator. Resulting concordance rate was 99.7%.

### Statistical analysis

Quality of SNP genotypes was assessed by study-wise call rate and exact test for Hardy-Weinberg disequilibrium (HWE) in patients and controls. We compared the results for genotype frequencies of the different polymorphisms between patient cohorts and control populations with the Chi-square test and logistic binary regression. All other models (dominant, recessive, allele frequencies) were computed by using two-tailed Fisher's Exact test. P-values were calculated using IBM SPSS Statistics 25 and GraphPad Prism 5. A p-value of less than 0.05 was considered to be significant. When a significant or nominal significant association was found, at least an extended or one replication cohort was screened.

## Results

### No study-wide significant association of GLO1 SNPs with ACP

We identified no significant association in logistic regression analysis and no significant difference in the genotype distribution in the ACP screening cohort compared to controls (**Table 2** and **Table C in S1 File**). As for *rs937662* the HWE was nominal significant in our controls (p = 0.047) and for both *rs937662* and *rs1699012* nominal significant results were observed in the NACP screening cohort in the dominant or recessive model (p = 0.08, respectively) we extended our analysis in the ACP cohort to 430 and 447 patients. Here, we found no association for both SNPs and no deviation from HWE for *rs937662* in the controls (p = 0.38; controls) (**Table 3**). Furthermore, genotype data of *rs1937780* displayed a borderline significance in the screening cohort (p = 0.08). Again, we extended our investigated groups and analysed *rs1937780* in a further German ACP cohort and additional European ACP patients from The Netherlands, Hungary, Italy, Romania and Poland. In the German cohort (n = 872) compared to controls (n = 1,474) the association was statistically significant (p = 0.003) as the genotype distribution between ACP patients and controls differed (GG: 40.0% vs. 33.1%; GA 45.9% vs. 51.6%; AA 14.1% vs. 15.3%) (**Table D in S1 File**). Of note, there was a deviation from HWE in the controls of the extended German cohort (p = 0.01). However, we did not find a significant association in logistic regression analysis (p = 0.134, odds ratio (OR) OR 1.117, 95% confidence interval (95% CI) 0.966–1.290) for this cohort. Otherwise, in the overall cohort of European ACP patients and controls we found no significant association in logistic regression analysis either (p = 0.214, OR 1.075, 95% CI 0.961–1.196) (**Table E in S1 File**). This result was confirmed for the corresponding genotype data and none of the five replication cohorts demonstrated a statistically significant association in the different genetic models used for computations (**Table 4** and **Tables D** and **E in S1 File**). Except for the named SNPs and SNP *rs2736655* (ACP patients p = 0.026), all SNPs corresponded to the HWE.

**Table 2:** Binary logistic regression results of the *GLO1* single nucleotide polymorphisms in patients with alcoholic and non-alcoholic chronic pancreatitis with covariate sex.

SNP	ACP			NACP		
	p-value	OR	95% CI	p-value	OR	95% CI
*rs2736655*	0.261	0.806	0.554–1.174	0.292	0.838	0.603–1.164
*rs9380765*	0.345	1.143	0.866–1.507	0.077	1.374	0.966–1.953
*rs13212218*	0.936	0.983	0.642–1.505	0.578	0.898	0.614–1.313
*rs937662*	0.621	0.952	0.783–1.157	0.070	1.207	0.985–1.480
*rs1621788*	0.548	1.085	0.831–1.417	0.526	1.083	0.847–1.384
*rs12198212*	0.483	1.116	0.822–1.514	0.216	1.196	0.901–1.589
*rs1699012*	0.508	0.935	0.767–1.140	0.009	0.763	0.623–0.933
*rs1616723*	0.639	1.127	0.684–1.855	0.962	1.012	0.630–1.623
*rs1937780*	0.134	1.117	0.966–1.290	0.112	1.176	0.963–1.436
*rs3778443*	0.992	1.003	0.564–1.783	0.323	1.348	0.746–2.438
*rs4746*	0.918	0.987	0.770–1.264	0.008	1.342	1.080–1.669
*rs17544798*	0.133	0.752	0.518–1.091	0.786	1.053	0.727–1.525

Abbreviations: SNP, single-nucleotide polymorphism; OR, Odds ratio; CI, confidence interval; ACP, alcoholic chronic pancreatitis; NACP, non-alcoholic chronic pancreatitis.

**Table 3:** Data of the analysed *GLO1* SNPs in patients with alcoholic chronic pancreatitis (ACP) and controls. For the calculations different genetic models were used.

SNP/Genetic model for calculation		p-value	OR	95% CI
**rs2736655**	**G/A**	0.36	0.849	0.613–1.176
	**GG + GA/AA**	0.19	0.778	0.538–1.124
	**GG/GA + AA**	0.48	2.034	0.407–10.18
**rs9380765**	**A/G**	0.61	1.074	0.838–1.377
	**AA + AG/GG**	0.32	1.218	0.828–1.794
	**AA/AG + GG**	0.91	0.971	0.627–1.505
**rs13212218**	**G/A**	1.00	0.991	0.675–1.453
	**GG + GA/AA**	0.83	0.944	0.623–1.430
	**GG/GA + AA**	0.65	2.777	0.308–25.03
**rs937662**	**C/T**	0.99	0.995	0.834–1.187
	**CC + CT/TT**	0.80	0.950	0.683–1.321
	**CC/ CT + TT**	0.89	1.021	0.787–1.324
**rs1621788**	**A/G**	0.54	1.087	0.853–1.385
	**AA + AG/GG**	0.76	1.089	0.724–1.639
	**AA/AG + GG**	0.49	1.143	0.778–1.679
**rs12198212**	**T/A**	0.37	1.136	0.864–1.494
	**TT + TA/AA**	0.87	1.108	0.567–2.164
	**TT/TA + AA**	0.33	1.192	0.845–1.682
**rs1699012**	**A/G**	0.32	0.909	0.759–1.089
	**AA + AG/GG**	0.33	0.878	0.680–1.133
	**AA/AG + GG**	0.65	0.942	0.730–1.215
**rs1616723**	**T/C**	0.81	0.942	0.590–1.503
	**TT + CT/CC**	1.00	1.390	0.125–15.44
	**TT/CT + CC**	0.79	0.921	0.561–1.512
**rs1937780**	**G/A**	0.61	1.073	0.834–1.381
	**GG + GA/AA**	0.30	0.763	0.466–1.251
	**GG/GA + AA**	0.15	1.324	0.927–1.890
**rs3778443**	**G/A**	0.49	0.813	0.479–1.381
	**GG + GA/AA**	1.00	1.952	0.079–48.18
	**GG/GA + AA**	0.39	0.776	0.447–1.344
**rs4746**	**T/G**	0.54	1.072	0.862–1.333
	**TT + TG/GG**	0.92	1.045	0.705–1.550
	**TT/TG + GG**	0.45	1.137	0.818–1.581
**rs17544798**	**A/T**	0.20	0.794	0.569–1.107
	**AA + AT/TT**	0.12	0.380	0.110–1.314
	**AA/AT + TT**	0.33	0.819	0.561–1.194

The different models comprise (order from top to bottom), allele frequencies, the dominant and the recessive model for computations. The number of patients and the genotype distribution of each variant are summarized in Table C **in S1 File**. Note: For *rs937662* and *rs1699012* the extended German cohorts have been used for computations. Calculations were performed using the Fisher’s exact test. Abbreviations: OR = odds ratio, 95% CI = 95% confidence interval.

**Table 4:** Data of the European alcoholic chronic pancreatitis (ACP) replication cohorts for rs1937780 in comparison to controls. Calculations were performed with different genetic models.

SNP/Genetic model for calculations		p-value	OR	95% CI
**rs1937780** **(Germany)**	**G/A**	**0.006**	1.187	1.051–1.340
	**GG + GA/AA**	0.46	1.103	0.870–1.399
	**GG/GA + AA**	**0.0009**	1.348	1.133–1.604
**rs1937780** **(Hungary)**	**G/A**	0.82	0.939	0.601–1.465
	**GG + GA/AA**	1.00	1.141	0.463–2.811
	**GG/GA + AA**	0.63	0.815	0.423–1.571
**rs1937780** **(The Netherlands)**	**G/A**	0.81	0.968	0.764–1.226
	**GG + GA/AA**	0.73	1.112	0.700–1.766
	**GG/GA + AA**	0.5	0.887	0.636–1.238
**rs1937780** **(Romania)**	**G/A**	0.45	1.293	0.717–2.333
	**GG + GA/AA**	0.34	2.121	0.513–8.767
	**GG/GA + AA**	0.66	1.286	0.539–3.068
**rs1937780** **(Poland)**	**G/A**	0.78	1.095	0.622–1.927
	**GG + GA/AA**	0.42	1.882	0.582–6.088
	**GG/GA + AA**	0.84	0.855	0.376–1.946
**rs1937780** **(Italy)**	**G/A**	0.49	1.141	0.815–1.599
	**GG + GA/AA**	0.43	1.298	0.713–2.364
	**GG/GA+AA**	0.70	1.126	0.673–1.883
**rs1937780** **(all)**	**G/A**	0.05	1.103	1.003–1.214
	**GG + GA/AA**	0.05	1.151	1.004–1.319
	**GG/GA + AA**	0.24	1.122	0.931–1.351

The different models comprise (order from top to bottom), allele frequencies, the dominant and the recessive model for computations. The number of patients and the genotype distribution of each variant are summarized in Table D **in S1 File**. Note, the German cohort used here comprises the screening cohort and further samples. Calculations were performed using the Fisher’s exact test. Abbreviations: OR = odds ratio, 95% CI = 95% confidence interval.

### No study-wide significant association of GLO1 SNPs with NACP

In the screening cohort the SNPs *rs937662*, *rs1699012*, and *rs4746* displayed a nominal significant difference in the recessive or dominant model (p = 0.08, 0.08, and 0.07, respectively) (**Table 5**). Otherwise, no differences in the genotype distributions were observed (**Table F in S1 File**). Furthermore, we detected a significant association for SNP *rs1699012* (p = 0.009, OR 0.763, 95% CI 0.623–0.933) and *rs4746* (p = 0.008, OR 1.342, 95% CI 1.080–1.669) in logistic regression analysis (**Table 2**). To elucidate a potential association, we extended the German NACP cohort and found no statistically significant association of the genotype distribution for *rs937662* (p = 0.15), whereas *rs1699012* and *rs4746* still displayed a significant association compared to controls (p = 0.02 and p = 0.008, respectively) (**Table G in S1 File**). Therefore, we investigated *rs4746* in an independent German (n = 450, patients; n = 581, controls), French (n = 767, patients; n = 1,049, controls), and Chinese (n = 928, patients; n = 1,008, controls) NACP cohort. Here, no association was found in all three replication cohorts (**Table 6**; for genotype distribution see **Table G in S1 File**). Finally, for SNP *rs1699012* we observed no significant association (p = 0.279, OR 0.903, 95% CI 0.750–1.087) in an independent German NACP cohort of 427 patients in logistic regression analysis (for different genetic models see **Table 6**). For the rare SNP *rs3778443* we observed a significant HWE (p = 0.002).

**Table 5:** Data of the analysed *GLO1* SNPs in patients with non-alcoholic chronic pancreatitis (NACP) and controls. For the calculations different genetic models were used.

SNP/Genetic model for calculations		p-value	OR	95% CI
**rs2736655**	**G/A**	0.35	0.853	0.614–1.184
	**GG + GA/AA**	0.11	0.423	0.150–1.218
	**GG/GA + AA**	0.63	0.909	0.624–1.324
**rs9380765**	**A/G**	0.31	0.874	0.682–1.119
	**AA + AG/GG**	0.44	0.844	0.551–1.294
	**AA/AG + GG**	0.35	0.807	0.536.1.217
**rs13212218**	**G/A**	0.56	0.889	0.610–1.295
	**GG + GA/AA**	1.00	0.900	0.199–4.065
	**GG/GA + AA**	0.53	0.875	0.579–1.322
**rs937662**	**C/T**	0.16	1.201	0.935–1.542
	**CC + CT/TT**	0.71	1.115	0.679–1.834
	**CC/ CT + TT**	0.08	1.388	0.967–1.993
**rs1621788**	**A/G**	0.62	1.070	0.838–1.366
	**AA + AG/GG**	0.84	1.057	0.702–1.592
	**AA/AG + GG**	0.55	1.128	0.766–1.663
**rs12198212**	**T/A**	0.24	1.182	0.898–1.563
	**TT + TA/AA**	0.48	1.385	0.677–2.833
	**TT/TA + AA**	0.29	1.207	0.854–1.705
**rs1699012**	**A/G**	0.12	0.813	0.631–1.048
	**AA + AG/GG**	0.08	0.650	0.402–1.051
	**AA/AG + GG**	0.37	0.842	0.589–1.204
**rs1616723**	**T/C**	0.91	0.974	0.608–1.560
	**TT + CT/CC**	1.00	1.390	0.125–15.44
	**TT/CT + CC**	0.90	0.956	0.581–1.575
**rs1937780**	**G/A**	0.32	1.145	0.882–1.486
	**GG + GA/AA**	0.39	1.336	0.749–2.380
	**GG/GA + AA**	0.45	1.161	0.803–1.680
**rs3778443**	**G/A**	0.37	1.341	0.729–2.470
	**GG + GA/AA**	0.56	0.319	0.029–3.541
	**GG/GA + AA**	0.27	1.519	0.790–2.920
**rs4746**	**T/G**	0.17	1.194	0.931–1.531
	**TT + TG/GG**	0.82	1.076	0.680–1.702
	**TT/TG + GG**	0.07	1.415	0.978–2.047
**rs17544798**	**A/T**	0.17	1.194	0.931–1.531
	**AA + AT/TT**	1.00	1.329	0.241–7.324
	**AA/AT + TT**	0.77	1.066	0.719–1.579

The different models comprise (order from top to bottom), allele frequencies, the dominant and the recessive model for computations. The number of patients and the genotype distribution of each variant are summarized in Table F **in S1 File**. Calculations were performed using the Fisher’s exact test. Abbreviations: OR = odds ratio, 95% CI = 95% confidence interval.

**Table 6:** Data of the replication cohorts of *GLO1* SNPs *rs937662*, *rs1699012*, *rs4746* in patients with non-alcoholic chronic pancreatitis (NACP) and controls. For computations different genetic models were used.

SNP/Genetic model for calculations		p-value	OR	95% CI
**rs937662**	**C/T**	0.06	1.212	0.994–1.477
	**CC + CT/ TT**	0.25	1.279	0.865–1.892
	**CC/CT + TT**	0.07	1.299	0.980–1.722
**rs1699012**	**A/G**	0.01	0.766	0.627–0.936
	**AA + AG/GG**	0.01	0.607	0.413–0.893
	**AA/AG + GG**	0.08	0.768	0.579–1.018
**rs1699012** **(Germany replication)**	**A/G**	0.31	0.907	0.755–1.089
	**AA + AG/GG**	0.77	0.938	0.640–1.375
	**AA/AG + GG**	0.22	0.854	0.664–1.099
**rs4746** **(Germany)**	**T/G**	**0.003**	1.360	1.108–1.669
	**TT + TG/GG**	0.09	1.407	0.952–2.079
	**TT/TG + GG**	**0.004**	1.571	1.168–2.112
**rs4746** **(Germany replication)**	**T/G**	0.34	1.093	0.917–1.303
	**TT + TG/GG**	0.63	1.097	0.797–1.510
	**TT/TG + GG**	0.31	1.152	0.883–1.503
**rs4746** **(France)**	**T/G**	0.11	1.117	0.979–1.276
	**TT + TG/GG**	0.06	1.262	0.998–1.596
	**TT/TG + GG**	0.44	1.087	0.888–1.331
**rs4746** **(China)**	**T/G**	0.27	1.175	0.897–1.539
	**TT + TG/GG**	0.81	0.815	0.312–2.132
	**TT/TG + GG**	0.25	0.837	0.621–1.129

The different models comprise (order from top to bottom), allele frequencies, the dominant and the recessive model for computations. The number of patients and the genotype distribution of each variant are summarized in Table G **in S1 File**. Note, for *rs937662*, *rs1699012*, and *rs4746* the extended German cohorts comprise the screening cohort and further German samples. For *rs1699012* the replication cohort from Munich is displayed. Calculations were performed using the Fisher’s exact test. Abbreviations: OR = odds ratio, 95% CI = 95% confidence interval.

## Discussion

There is a biological plausibility that GLO1 is relevant in inflammatory processes and as such for the development of CP, although conflicting results have been reported in other diseases thus far. In this work, we investigated a potential genetic association of *GLO1* variants with ACP and NACP. We failed to identify an association between CP and one functionally relevant variant and 11 tagging SNPs covering the *GLO1* locus.

In our German ACP patients (total cohort) *rs1937780* genotypes differed significantly between patients and controls (p = 0.003) and significance was also observed in the dominant model. Contrary, we found neither an association nor a comparable trend in the distinct European cohorts. The same variant has recently been investigated in pancreatic cancer patients and an association was absent [33]. As functional consequences of this variant are unknown and overall genetic data are statistically not significant, it is unlikely that *rs1937780* plays a prominent role in CP development.

In the NACP cohort the three variants *rs937662*, *rs1699012*, and *rs4746* showed borderline significant results in distinct analysis models that, however, were not confirmed in the extended or replication cohorts. For the variants *rs937662* and *rs1699012* no prior clinical relevance has been reported and therefore an association with CP is again unlikely. Contrary, *rs4746* was associated with a wide spectrum of disorders ranging from diabetes [19], atherosclerosis [20], chronic renal failure [21], vascular diseases [22,34], neuropsychiatric disorders [24,25], and even to cancer [26–29]. In our work the borderline significance of the screening cohort was disproved in three large NACP cohorts from Germany, France, and China. As such, although, a functional relevance for this variant has been reported, our data show no association with CP.

We investigated cohorts with a reasonable number of patients and extended these cohorts whenever statistically or nominal significant results were obtained. Therefore, a prominent disease association of *GLO1* variants with CP can be ruled out with high certainty. Nevertheless, our approach is not capable of identifying rare associating variants. We therefore analyzed whole exome sequencing data from an ongoing project but did not identify rare variants enriched in our NACP patients (unpublished data). Nonetheless, our study is limited in its restriction to a Caucasian (German) cohort as only one SNP was analyzed in an Asian replication cohort and therefore we may have missed specific associations in other ethnicities.

In summary, we performed a comprehensive investigation of *GLO1* variants and did not demonstrate a prominent role for CP development in alcoholic and the non-alcoholic etiologies of the disease.

## Supporting information

S1 File **Figure A. Linkage disequilibrium figure of the *GLO1* locus generated by the SNPinfo LD TAG SNP Selection tool**.
To generate the linkage disequilibrium (LD) figure, the SNPinfo LD TAG SNP Selection tool (https://snpinfo.niehs.nih.gov/snpinfo/snptag.html) with the following parameters was applied: LD threshold of 0.8; a minimum of one SNP tagged; a minimum of 5 valid genotypes to calculate LD in populations with European ancestry (CEU); integrated region with 10.000 bp in the 5’-region and the 3’-region of GLO1. Abbreviations: SNP, single nucleotide polymorphism; LD, linkage disequilibrium; CEU, Northern Europeans from Utah. For the following SNPs we used tagging SNPs in our study: rs10484854 was tagged by rs12198212; rs1781735 by rs1621788; rs6458064 by rs937662; and rs9394523 by rs13212218. As demonstrated in the figure the tagging SNPs represented the depicted haplotypes. In Table A in S1 File the information on the SNPs selected according to the published literature and by SNPinfo is summarized.
**Table A. Overview of screened *GLO1* SNPs that were identified by SNPinfo or by a literature research**.
We used the SNPinfo LD TAG SNP Selection tool to identify SNPs in the GLO1 locus that cover the haplotypes of the gene. As several variants have been reported in the literature, we included these using tagging SNPs, where possible. The corresponding literature for the screened SNPs is indicated in brackets. For rs4746 several studies reported associations and functional data are available in addition. * These SNPs have been tagged by the screened SNP rs1616723, rs9380765, rs13212218, and rs1621788 respectively.
**Table B**. Polymerase chain reaction (PCR) primers and probes for melting curve analysis of all *GLO1* SNPs. Abbreviations: fw, forward; rv, reverse; XI, internal dye modified base; LC610, 5´- LightCycler Red 610; LC640 (sensor probe), LightCycler Red 640 (sensor probe); FL, 3'-Fluorescein labelling (anchor probe); PH, 3´-phosphate.
**Table C. Genotype data of the analysed *GLO1* SNPs in patients with alcoholic chronic pancreatitis (ACP) and controls**.
Note: For rs937662 and rs1699012 the extended German cohorts are shown. Calculations were performed using the Chi-square test (two-sided). Abbreviations: Contr. = controls, Pat. = patients.
**Table D. Genotype data of the GLO1 SNP rs1937780 in patients with alcoholic chronic pancreatitis in European cohorts including Germany**. Note: The German cohort comprises the screening cohort and additional samples. Calculations were performed using the Chi-square test (two-sided). Abbreviations: Contr. = controls, Pat. = patients.
**Table E. Results of logistic regression with covariate gender for the GLO1 SNP rs1937780 in patients with alcoholic chronic pancreatitis in European cohorts including Germany**.
Note: The German cohort comprises the screening cohort and additional samples. Calculations were performed using logistic regression. Abbreviations: OR, Odds ratio; CI, confidence interval; ACP, alcoholic chronic pancreatitis.
**Table F. Genotype data of the analysed GLO1 SNPs in German patients with non-alcoholic chronic pancreatitis (NACP) and controls**.
Calculations were performed using the Chi-square test (two-sided). Abbreviations: Contr. = controls, Pat. = patients.
**Table G. Genotype data of the analysed GLO1 SNPs rs937662, rs1699012, rs4746 in the extended NACP cohorts**.
Note: For rs937662, rs1699012, and rs4746 the extended German cohorts comprise the screening cohort and additional German samples. Calculations were performed using the Chi-square test (two-sided). Abbreviations: Contr. = controls, Pat. = patients. ‖ NACP replication cohort and controls from Germany. $ Independent NACP replication cohort from Munich. § NACP cohort and controls from France. € NACP cohort and controls from China.
(DOCX)

## Abbreviations

ACP: alcoholic chronic pancreatitis
CI: confidence interval
CP: chronic pancreatitis
GLO1: Glyoxalase-
HWE: Hardy-Weinberg-disequilibrium
NACP: non-alcoholic chronic pancreatitis
OR: odds ratio
PCR: polymerase chain reaction
ROS: reactive oxygen species
SNP: single nucleotide polymorphism
