# Supplementary material for: Common variants in glyoxalase I do not increase chronic pancreatitis risk
Source: PLoS One. 2019 Oct 29;14(10):e0222927. doi: 10.1371/journal.pone.0222927 (PMC6818803; doi:10.1371/journal.pone.0222927)
Supplement: S1 File — Figure A. Linkage disequilibrium figure of the GLO1 locus generated by the SNPinfo LD TAG SNP Selection tool. To generate the linkage disequilibrium (LD) figure, the SNPinfo LD TAG SNP Selection tool (https://snpinfo.niehs.nih.gov/snpinfo/snptag.html) with the following parameters was applied: LD threshold of 0.8; a minimum of one SNP tagged; a minimum of 5 valid genotypes to calculate LD in populations with European ancestry (CEU); integrated region with 10.000 bp in the 5’-region and the 3’-region of GLO1. Abbreviations: SNP, single nucleotide polymorphism; LD, linkage disequilibrium; CEU, Northern Europeans from Utah. For the following SNPs we used tagging SNPs in our study: rs10484854 was tagged by rs12198212; rs1781735 by rs1621788; rs6458064 by rs937662; and rs9394523 by rs13212218. As demonstrated in the figure the tagging SNPs represented the depicted haplotypes. In Table A in S1 File the information on the SNPs selected according to the published literature and by SNPinfo is summarized. Table A. Overview of screened GLO1 SNPs that were identified by SNPinfo or by a literature research. We used the SNPinfo LD TAG SNP Selection tool to identify SNPs in the GLO1 locus that cover the haplotypes of the gene. As several variants have been reported in the literature, we included these using tagging SNPs, where possible. The corresponding literature for the screened SNPs is indicated in brackets. For rs4746 several studies reported associations and functional data are available in addition. * These SNPs have been tagged by the screened SNP rs1616723, rs9380765, rs13212218, and rs1621788 respectively. Table B. Polymerase chain reaction (PCR) primers and probes for melting curve analysis of all GLO1 SNPs. Abbreviations: fw, forward; rv, reverse; XI, internal dye modified base; LC610, 5´- LightCycler Red 610; LC640 (sensor probe), LightCycler Red 640 (sensor probe); FL, 3'-Fluorescein labelling (anchor probe); PH, 3´-phosphate. Table C. Genotype data of the analysed G [file pone.0222927.s001.docx]

**Supporting Information**

**Title:** Common variants in glyoxalase I do not increase chronic pancreatitis risk

**Figure A:** Linkage disequilibrium figure of the *GLO1* locus generated by the SNPinfo LD TAG SNP Selection tool.


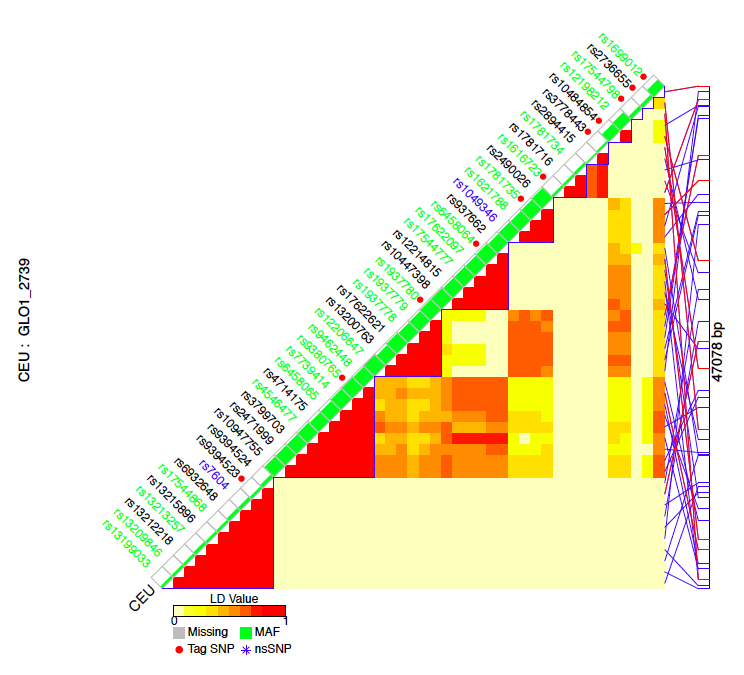


To generate the linkage disequilibrium (LD) figure, the SNPinfo LD TAG SNP Selection tool (<https://snpinfo.niehs.nih.gov/snpinfo/snptag.html>https://snpinfo.niehs.nih.gov/snpinfo/snptag.html) with the following parameters was applied: LD threshold of 0.8; a minimum of one SNP tagged; a minimum of 5 valid genotypes to calculate LD in populations with European ancestry (CEU); integrated region with 10.000 bp in the 5’-region and the 3’-region of *GLO1*. Abbreviations: SNP, single nucleotide polymorphism; LD, linkage disequilibrium; CEU, Northern Europeans from Utah. For the following SNPs we used tagging SNPs in our study: *rs10484854* was tagged by *rs12198212*; *rs1781735* by *rs1621788*; *rs6458064* by *rs937662*; and *rs9394523* by *rs13212218*. As demonstrated in the Figure the tagging SNPs represented the depicted haplotypes. In Supplementary Table A the information on the SNPs selected according to the published literature and by SNPinfo is summarized.

**Table A**: Overview of screened *GLO1* SNPs that were identified by SNPinfo or by a literature research.

| **Screened SNP** | **Tagging SNP** | **Identified by** | **Literature** |
| --- | --- | --- | --- |
|  |  |  |  |
| rs1699012 |  | SNPinfo | None available |
| rs1616723 | rs1781716 *, rs1781734 * | SNPinfo/Literature | Yes [1] |
| rs1937780 |  | SNPinfo | Yes [2] |
| rs2736655 |  | SNPinfo | Yes [1-3] |
| rs3778443 |  | SNPinfo | None available |
| rs4746 |  | Literature | Yes [4-24] |
| rs17544798 |  | SNPinfo | None available |
| rs9380765 | rs10947755 *, rs4714175 * | SNPinfo/Literature | Yes [1] |
| rs13212218 | rs9394523, rs1130534 * | SNPinfo | Yes [25] |
| rs937662 | rs6458064 | SNPinfo | Yes [1] |
| rs12198212 | rs10484854 | SNPinfo | Yes [1,17] |
| rs1621788 | rs1781735, rs1049346 * | SNPinfo/Literature | Yes [3,25] |

We used the SNPinfo LD TAG SNP Selection tool to identify SNPs in the *GLO1* locus that cover the haplotypes of the gene. As several variants have been reported in the literature, we included these using tagging SNPs, where possible. The corresponding literature for the screened SNPs is indicated in brackets. For *rs4746* several studies reported associations and functional data are available in addition. * These SNPs have been tagged by the screened SNP *rs1616723*, *rs9380765*, *rs13212218*, and *rs1621788* respectively.

**Table B**: Polymerase chain reaction (PCR) primers and probes for melting curve analysis of all *GLO1* SNPs.

| **SNP** | **Sequence primer and probes (5´- 3´)** | **Annealing temperature** |
| --- | --- | --- |
| *rs1699012* | CCAGTAATAGAGCAGTTTCACAATCT (fw) | 62°C |
|  | CCCACGATCCTGAGTATTAAGTGTC (rv) |  |
|  | TACTGAACCGGTAAXITCAGGGAA--PH |  |
| *rs1616723* | CATTACAAGGGACGTATACATGGC (fw) | 62°C |
|  | TCCTATGGTTGGACATTTAGCCT (rv) |  |
|  | CATTTATTTTCCCTAATGAACAXITCATC--PH |  |
| *rs1937780* | GTTTTGCCTTTTCACATTTATGTCT (fw) | 60°C |
|  | TCCAGAACTACCTAGCTGGACC (rv) |  |
|  | AGGAAAACGTAAATCCAXIAGAGGATTATAG--PH |  |
| *rs2736655* | TGGGCTAACATGGTGAAACCCT (fw) | 62°C |
|  | TCAAGGCTATAGTGAGCTATGACAGT (rv) |  |
|  | TGAGGACAGAGTGAXIAGCAAATTCCTT--PH |  |
| *rs3778443* | ATCTAGAGAGTGTTTTCAGACCCATC (fw) | 58°C |
|  | TGCAAGATCAGTGGATATGACCT (rv) |  |
|  | CCCTTXITCAGGCTGGCTGAGATAGAG--PH |  |
| *rs4746* | GTTAGGCCAATTATGTAAAGTAGCAT (fw) | 62°C |
|  | CGAGACTCTGAAATTAAACAACTCC (rv) |  |
|  | GCACTGAXIAGATGATGCGACCCAGA--PH |  |
| *rs17544798* | TGCCTCAAACTAGTCCTTCAGG (fw) | 58°C |
|  | GGGTATGCACAGCATGATCG (rv) |  |
|  | GAATATTAAAATCTAXITGGAAGCAAAGTGC--PH |  |
| *rs9380765* | CTTCATGGGCTTTTACTTACCTGT (fw) | 58°C |
|  | ACGATCAAGTTCCATTTAGTGACATG (rv) |  |
|  | TTGTTTCTTTTGTTGTTGTTGTXITAAGTTGA--PH |  |
| *rs13212218* | ACTGCCCTTGTTTACTGGTAAG (fw) | 62°C |
|  | CGGGTGACTGTTTTAACTTGG (rv) |  |
|  | CCCTCTCACTTGTTCAGATG—FL |  |
|  | LC610-CCTTACCAGTAAACAAGGGCAGTGC--PH |  |
| *rs937662* | ACTGCCCTTGTTTACTGGTAAG (fw) | 62°C |
|  | CGGGTGACTGTTTTAACTTGG (rv) |  |
|  | CAGTGAGGGAGGAACAAG--FL |  |
|  | LC640-GCAAAACTCTGTTCTCTCGCAGCC--PH |  |
| *rs12198212* | GTTTGACCCACCTGGGATA (fw) | 62°C |
|  | CATGACCTGTGAATGTGACCTTATT (rv) |  |
|  | CCTTGAGAATTTTTACTTCATTAC--FL |  |
|  | LC640-TCTTCAAAGATTTTTTTTTTCCAAATAAGG--PH |  |
| *rs1621788* | GTTTGACCCACCTGGGATA (fw) | 62°C |
|  | CATGACCTGTGAATGTGACCTTATT (rv) |  |
|  | CTGGTTCCCGCTAGACCTT--FL |  |
|  | LC610-GACTCCGCTGGACTTTGTAGCCCC--PH |  |

Abbreviations: fw, forward; rv, reverse; XI, internal dye modified base; LC610, 5´- LightCycler Red 610; LC640 (sensor probe), LightCycler Red 640 (sensor probe); FL, 3'-Fluorescein labelling (anchor probe); PH, 3´-phosphate.

**Table C:** Genotype data of the analysed *GLO1* SNPs in patients with alcoholic chronic pancreatitis (ACP) and controls.

| **Country** | **SNP** | **ID** | **Genotype** | | | **p-value** |
| --- | --- | --- | --- | --- | --- | --- |
| **Germany** | **rs2736655** |  | **GG** | **GA** | **AA** |  |
|  |  | Pat. | 144/219 (65.8 %) | 73/219 (33.3 %) | 2/219 (0.9 %) | 0.21 |
|  |  | Contr. | 232/326 (71.2 %) | 88/326 (27 %) | 6/326 (1.8 %) |  |
|  | **rs9380765** |  | **AA** | **AG** | **GG** |  |
|  |  | Pat. | 62/204 (30.4 %) | 101/204 (49.5 %) | 41/204 (20.1 %) | 0.54 |
|  |  | Contr. | 86/326 (26.4 %) | 176/326 (54 %) | 64/326 (19.6 %) |  |
|  | **rs13212218** |  | **GG** | **GA** | **AA** |  |
|  |  | Pat. | 170/219 (77.6 %) | 48/219 (21.9 %) | 1/219 (0.5 %) | 0.58 |
|  |  | Contr. | 250/318 (78.6 %) | 64/318 (20.1 %) | 4/318 (1.3 %) |  |
|  | **rs937662** |  | **CC** | **CT** | **TT** |  |
|  |  | Pat. | 146/430 (34.0 %) | 211/430 (49.1 %) | 73/430 (17.0 %) | 0.92 |
|  |  | Contr. | 208/621 (33.5 %) | 312/621 (50.2 %) | 101/621 (16.3 %) |  |
|  | **rs1621788** |  | **AA** | **AG** | **GG** |  |
|  |  | Pat. | 63/221 (28.5 %) | 109/221 (49.3 %) | 49/221 (22.2 %) | 0.77 |
|  |  | Contr. | 83/321 (25.9 %) | 162/321 (50.5 %) | 76/321 (23.7 %) |  |
|  | **rs12198212** |  | **TT** | **TA** | **AA** |  |
|  |  | Pat. | 121/220 (55 %) | 84/220 (38.2 %) | 15/220 (6.8 %) | 0.60 |
|  |  | Contr. | 162/320 (50.6 %) | 134/320 (41.9 %) | 24/320 (7.5 %) |  |
|  | **rs1699012** |  | **AA** | **AG** | **GG** |  |
|  |  | Pat. | 177/447 (39.6 %) | 214/447 (47.9 %) | 56/447 (12.5 %) | 0.55 |
|  |  | Contr. | 261/608 (42.9 %) | 277/608 (45.6 %) | 70/608 (11.5 %) |  |
|  | **rs1616723** |  | **TT** | **TC** | **CC** |  |
|  |  | Pat. | 181/213 (85 %) | 31/213 (14.6 %) | 1/213 (0.5 %) | 0.90 |
|  |  | Contr. | 264/307 (86 %) | 41/307 (13.4 %) | 2/307 (0.7 %) |  |
|  | **rs1937780** |  | **GG** | **GA** | **AA** |  |
|  |  | Pat. | 89/215 (41.4%) | 92/215 (42.8%) | 34/215 (15.8%) | 0.08 |
|  |  | Contr. | 111/319 (34.8%) | 168/319 (52.7%) | 40/319 (12.5%) |  |
|  | **rs3778443** |  | **GG** | **GA** | **AA** |  |
|  |  | Pat. | 184/210 (87.6 %) | 26/210 (12.4 %) | 0/210 (0 %) | 0.43 |
|  |  | Contr. | 292/324 (90.1 %) | 31/324 (9.6 %) | 1/324 (0.3 %) |  |
|  | **rs4746** |  | **TT** | **TG** | **GG** |  |
|  |  | Pat. | 74/230 (32.2 %) | 114/230 (49.6 %) | 42/230 (18.3 %) | 0.75 |
|  |  | Contr. | 171/581 (29.4%) | 300/581 (51.6%) | 110/581 (18.9 %) |  |
|  | **rs17544798** |  | **AA** | **AT** | **TT** |  |
|  |  | Pat. | 151/219 (68.9 %) | 61/219 (27.9 %) | 7/219 (3.2 %) | 0.20 |
|  |  | Contr. | 236/323 (73.1 %) | 83/323 (25.7 %) | 4/323 (1.2 %) |  |

Note: For *rs937662* and *rs1699012* the extended German cohorts are shown. Calculations were performed using the Chi-square test (two-sided). Abbreviations: Contr.=controls, Pat.=patients.

**Table D:** Genotype data of the *GLO1* SNP *rs1937780* in patients with alcoholic chronic pancreatitis in European cohorts including Germany.

| **Country** | **ID** | **GG** | **GA** | **AA** | **p-value** |
| --- | --- | --- | --- | --- | --- |
| **Germany** |  |  |  |  |  |
|  | Pat. | 349/872 (40.0 %) | 400/872 (45.9 %) | 123/872 (14.1 %) | **0.003** |
|  | Contr. | 488/1474 (33.1 %) | 760/1474 (51.6 %) | 226/1474 (15.3 %) |  |
| **Hungary** |  |  |  |  |  |
|  | Pat. | 17/53 (32.1 %) | 29/53 (54.7 %) | 7/53 (13.2%) | 0.73 |
|  | Contr. | 62/169 (36.7 %) | 82/169 (48.5 %) | 25/169 (14.8 %) |  |
| **The Netherlands** |  |  |  |  |  |
|  | Pat. | 84/223 (37.7 %) | 108/223 (48.4 %) | 31/223 (13.9 %) | 0.60 |
|  | Contr. | 173/427 (40.5 %) | 189/427 (44.3 %) | 65/427 (15.2 %) |  |
| **Romania** |  |  |  |  |  |
|  | Pat. | 15/43 (34.9 %) | 25/43 (58.1 %) | 3/43 (7.0 %) | 0.54 |
|  | Contr. | 15/51 (29.4 %) | 29/51 (56.9 %) | 7/51 (13.7 %) |  |
| **Poland** |  |  |  |  |  |
|  | Pat. | 12/36 (33.3 %) | 20/36 (55.6 %) | 4/36 (11.1 %) | 0.42 |
|  | Contr. | 31/84 (36.9 %) | 37/84 (44.0 %) | 16/84 (19.0 %) |  |
| **Italy** |  |  |  |  |  |
|  | Pat. | 69/214 (32.2 %) | 109/214 (50.9 %) | 36/214 (16.8 %) | 0.68 |
|  | Contr. | 30/101 (29.7 %) | 50/101 (49.5 %) | 21/101 (20.8 %) |  |
| **All** |  |  |  |  |  |
|  | Pat. | 546/1441 (37.9 %) | 691/1441 (48.0 %) | 204/1441 (14.2 %) | 0.11 |
|  | Contr. | 799/2306 (34.6 %) | 1147/2306 (49.7 %) | 360/2306 (15.6 %) |  |

Note: The German cohort comprises the screening cohort and additional samples. Calculations were performed using the Chi-square test (two-sided). Abbreviations: Contr.=controls, Pat.=patients.

**Table E:** Results of logistic regression with covariate gender for the *GLO1* SNP *rs1937780* in patients with alcoholic chronic pancreatitis in European cohorts including Germany.

| **Country** | **p-value** | **OR** | **95 % CI** |
| --- | --- | --- | --- |
| Germany | 0.134 | 1.117 | 0.966-1.290 |
| Hungary | 0.787 | 0.938 | 0.592-1.487 |
| The Netherlands | 0.761 | 1.041 | 0.805-1.345 |
| Romania | 0.452 | 1.316 | 0.643-2.691 |
| Poland | 0.992 | 1.003 | 0.532-1.891 |
| Italy | 0.808 | 1.182 | 0.808-1.730 |
| **All** | **0.214** | **1.072** | **0.961-1.196** |

Note: The German cohort comprises the screening cohort and additional samples. Calculations were performed using logistic regression. Abbreviations: OR, Odds ratio; CI, confidence interval; ACP, alcoholic chronic pancreatitis.

**Table F:** Genotype data of the analysed *GLO1* SNPs in German patients with non-alcoholic chronic pancreatitis (NACP) and controls.

| **Country** | **SNP** | **ID** | **Genotype** | | | | **p-value** | |
| --- | --- | --- | --- | --- | --- | --- | --- | --- |
| **Germany** | **rs2736655** |  | **GG** | **GA** | **AA** |  | |  |
|  |  | Pat. | 148/214 (69.2 %) | 57/214 (26.6 %) | 9/214 (4.2 %) | 0.26 | |  |
|  |  | Contr. | 232/326 (71.2 %) | 88/326 (27 %) | 6/326 (1.8 %) |  |  |  |
|  | **rs9380765** |  | **AA** | **AG** | **GG** |  | |  |
|  |  | Pat. | 46/205 (22.4 %) | 113/205 (55.1 %) | 46/205 (22.4 %) | 0.52 | |  |
|  |  | Contr. | 86/326 (26.4 %) | 176/326 (54 %) | 64/326 (19.6 %) |  |  |  |
|  | **rs13212218** |  | **GG** | **GA** | **AA** |  | |  |
|  |  | Pat. | 164/215 (76.3 %) | 48/215 (22.3 %) | 3/215 (1.4 %) | 0.82 | |  |
|  |  | Contr. | 250/318 (78.6 %) | 64/318 (20.1 %) | 4/318 (1.3 %) |  |  |  |
|  | **rs937662** |  | **CC** | **CT** | **TT** |  | |  |
|  |  | Pat. | 83/215 (38.6%) | 103/215 (47.9%) | 29/215 (13.5%) | 0.20 | |  |
|  |  | Contr. | 101/324 (31.2%) | 175/324 (54%) | 48/324 (14.8%) |  |  |  |
|  | **rs1621788** |  | **AA** | **AG** | **GG** |  | |  |
|  |  | Pat. | 61/216 (28.2 %) | 106/216 (49.1 %) | 49/216 (22.7 %) | 0.83 | |  |
|  |  | Contr. | 83/321 (25.9 %) | 162/321 (50.5 %) | 76/321 (23.7 %) |  |  |  |
|  | **rs12198212** |  | **TT** | **TA** | **AA** |  | |  |
|  |  | Pat. | 120/217 (55.3 %) | 85/217 (39.2 %) | 12/217 (5.5 %) | 0.47 | |  |
|  |  | Contr. | 162/320 (50.6 %) | 134/320 (41.9 %) | 24/320 (7.5 %) |  |  |  |
|  | **rs1699012** |  | **AA** | **AG** | **GG** |  | |  |
|  |  | Pat. | 80/212 (37.7%) | 93/212 (43.9%) | 39/212 (18.4%) | 0.20 | |  |
|  |  | Contr. | 131/313 (41.9%) | 142/313 (45.4%) | 40/313 (12.8%) |  |  |  |
|  | **rs1616723** |  | **TT** | **TC** | **CC** | 0.93 | |  |
|  |  | Pat. | 182/213 (85.4 %) | 30/213 (14.1 %) | 1/213 (0.5 %) |  |  |  |
|  |  | Contr. | 264/307 (86 %) | 41/307 (13.4 %) | 2/307 (0.7 %) |  |  |  |
|  | **rs1937780** |  | **GG** | **GA** | **AA** |  | |  |
|  |  | Pat. | 75/196 (38.3%) | 102/196 (52%) | 19/196 (9.7%) | 0.53 | |  |
|  |  | Contr. | 111/319 (34.8%) | 168/319 (52.7%) | 40/319 (12.5%) |  |  |  |
|  | **rs3778443** |  | **GG** | **GA** | **AA** |  | |  |
|  |  | Pat. | 194/208 (93.3 %) | 12/208 (5.8 %) | 2/208 (1 %) | 0.19 | |  |
|  |  | Contr. | 292/324 (90.1 %) | 31/324 (9.6 %) | 1/324 (0.3 %) |  |  |  |
|  | **rs4746** |  | **TT** | **TG** | **GG** |  | |  |
|  |  | Pat. | 78/214 (36.4%) | 100/214 (46.7%) | 36/214 (16.8%) | 0.17 | |  |
|  |  | Contr. | 92/318 (28.8%) | 170/318 (53.3%) | 57/318 (17.9%) |  |  |  |
|  | **rs17544798** |  | **AA** | **AT** | **TT** |  | |  |
|  |  | Pat. | 159/214 (74.3 %) | 53/214 (24.8 %) | 2/214 (0.9 %) | 0.92 | |  |
|  |  | Contr. | 236/323 (73.1 %) | 83/323 (25.7 %) | 4/323 (1.2 %) |  |  |  |

Calculations were performed using the Chi-square test (two-sided). Abbreviations: Contr.=controls, Pat.=patients.

**Table G:** Genotype data of the analysed *GLO1* SNPs *rs937662*, *rs1699012*, *rs4746* in the extended NACP cohorts.

| **SNP** | **ID** | **Genotype** | | | **p-value** |
| --- | --- | --- | --- | --- | --- |
| **rs937662** |  | **CC** | **CT** | **TT** |  |
|  | Pat. (NACP) | 123/311 (39.5 %) | 147/311 (47.3 %) | 41/311 (13.2 %) | 0.15 |
|  | Contr. | 208/621 (33.5 %) | 312/621 (50.2 %) | 101/621 (16.3 %) |  |
| **rs1699012** |  | **AA** | **AG** | **GG** |  |
|  | Pat. (NACP) | 112/306 (36.6 %) | 140/306 (45.8 %) | 54/306 (17.6 %) | 0.02 |
|  | Contr. | 261/608 (42.9 %) | 277/608 (45.6 %) | 70/608 (11.5 %) |  |
|  |  | **AA** | **AG** | **GG** | 0.47 |
|  | Pat. (NACP)^$^ | 167/427 (39.1 %) | 208/427 (48.7 %) | 52/427 (12.2 %) |  |
|  | Contr. | 261/608 (42.9 %) | 277/608 (45.6 %) | 70/608 (11.5 %) |  |
| **rs4746** |  | **TT** | **TG** | **GG** |  |
|  | Pat. (NACP) | 114/288 (39.6 %) | 133/288 (46.2 %) | 41/288 (14.2 %) | 0.008 |
|  | Contr. | 171/581 (29.4 %) | 300/581 (51.6 %) | 110/581 (18.9 %) |  |
|  |  | **TT** | **TG** | **GG** |  |
|  | Pat. (NACP)^‖^ | 146/450 (32.4 %) | 225/450 (50.0 %) | 79/450 (17.6 %) | 0.56 |
|  | Contr.^‖^ | 171/581 (29.4 %) | 300/581 (51.6 %) | 110/581 (18.9 %) |  |
|  |  | **TT** | **TG** | **GG** |  |
|  | Pat. (NACP)^§^ | 241/767 (31.4 %) | 387/767 (50.5 %) | 139/767 (18.1) | 0.15 |
|  | Contr.^§^ | 311/1049 (29.6 %) | 509/1049 (48.5 %) | 229/1049 (21.8 %) |  |
|  |  | **TT** | **TG** | **GG** |  |
|  | Pat. (NACP)^€^ | 349/464 (75.2 %) | 106/464 (22.8 %) | 9/464 (1.9%) | 0.81 |
|  | Contr.^€^ | 395/504 (78.4 %) | 101/504 (20.0 %) | 8/504 (1.6 %) |  |

Note: For *rs937662*, *rs1699012*, and *rs4746* the extended German cohorts comprise the screening cohort and additional German samples. Calculations were performed using the Chi-square test (two-sided). Abbreviations: Contr.=controls, Pat.=patients. ^‖^ NACP replication cohort and controls from Germany. ^$^ Independent NACP replication cohort from Munich. ^§^ NACP cohort and controls from France. ^€^ NACP cohort and controls from China

Reference List

1. Bangel FN, Yamada K, Arai M, Iwayama Y, Balan S, Toyota T, et al. Genetic analysis of the glyoxalase system in schizophrenia. Prog Neuropsychopharmacol Biol Psychiatry. 2015;59: 105-110

2. Duan Z, Chen G, Chen L, Stolzenberg-Solomon R, Weinstein SJ, Mannisto S, et al. Determinants of concentrations of N(epsilon)-carboxymethyl-lysine and soluble receptor for advanced glycation end products and their associations with risk of pancreatic cancer. Int J Mol Epidemiol Genet. 2014;5: 152-163

3. Donato L, Scimone C, Nicocia G, Denaro L, Robledo R, Sidoti A, et al. GLO1 gene polymorphisms and their association with retinitis pigmentosa: a case-control study in a Sicilian population. Mol Biol Rep. 2018;45: 1349-1355

4. Junaid MA, Kowal D, Barua M, Pullarkat PS, Sklower BS, Pullarkat RK. Proteomic studies identified a single nucleotide polymorphism in glyoxalase I as autism susceptibility factor. Am J Med Genet A. 2004;131: 11-17

5. Barua M, Jenkins EC, Chen W, Kuizon S, Pullarkat RK, Junaid MA. Glyoxalase I polymorphism rs2736654 causing the Ala111Glu substitution modulates enzyme activity--implications for autism. Autism Res. 2011;4: 262-270

6. Groener JB, Reismann P, Fleming T, Kalscheuer H, Lehnhoff D, Hamann A, et al. C332C genotype of glyoxalase 1 and its association with late diabetic complications. Exp Clin Endocrinol Diabetes. 2013;121: 436-439

7. Wu JC, Li XH, Wang JB, Tang JF, Wang YF, Peng YD. Glyoxalase I and aldose reductase gene polymorphisms and susceptibility to carotid atherosclerosis in type 2 diabetes. Genet Test Mol Biomarkers. 2011;15: 273-279

8. Kalousova M, Germanova A, Jachymova M, Mestek O, Tesar V, Zima T. A419C (E111A) polymorphism of the glyoxalase I gene and vascular complications in chronic hemodialysis patients. Ann N Y Acad Sci. 2008;1126: 268-271

9. Gale CP, Futers TS, Summers LK. Common polymorphisms in the glyoxalase-1 gene and their association with pro-thrombotic factors. Diab Vasc Dis Res. 2004;1: 34-39

10. Rinaldi C, Bramanti P, Fama A, Scimone C, Donato L, Antognelli C, et al. Glyoxalase I A111E, Paraoxonase 1 Q192R and L55M Polymorphisms in Italian Patients with Sporadic Cerebral Cavernous Malformations: a Pilot Study. J Biol Regul Homeost Agents. 2015;29: 493-500

11. Williams R, Lim JE, Harr B, Wing C, Walters R, Distler MG, et al. A common and unstable copy number variant is associated with differences in Glo1 expression and anxiety-like behavior. PLoS One. 2009;4: e4649

12. Sidoti A, Antognelli C, Rinaldi C, D'Angelo R, Dattola V, Girlanda P, et al. Glyoxalase I A111E, paraoxonase 1 Q192R and L55M polymorphisms: susceptibility factors of multiple sclerosis? Mult Scler. 2007;13: 446-453

13. Antognelli C, Mezzasoma L, Mearini E, Talesa VN. Glyoxalase 1-419C>A variant is associated with oxidative stress: implications in prostate cancer progression. PLoS One. 2013;8: e74014

14. Krechler T, Jachymova M, Mestek O, Zak A, Zima T, Kalousova M. Soluble receptor for advanced glycation end-products (sRAGE) and polymorphisms of RAGE and glyoxalase I genes in patients with pancreas cancer. Clin Biochem. 2010;43: 882-886

15. Antognelli C, Del BC, Ludovini V, Gori S, Talesa VN, Crino L, et al. CYP17, GSTP1, PON1 and GLO1 gene polymorphisms as risk factors for breast cancer: an Italian case-control study. BMC Cancer. 2009;9: 115

16. Chocholaty M, Jachymova M, Schmidt M, Havlova K, Krepelova A, Zima T, et al. Polymorphisms of the receptor for advanced glycation end-products and glyoxalase I in patients with renal cancer. Tumour Biol. 2015;36: 2121-2126

17. Engelen L, Ferreira I, Brouwers O, Henry RM, Dekker JM, Nijpels G, et al. Polymorphisms in glyoxalase 1 gene are not associated with vascular complications: the Hoorn and CoDAM studies. J Hypertens. 2009;27: 1399-1403

18. Chen F, Wollmer MA, Hoerndli F, Munch G, Kuhla B, Rogaev EI, et al. Role for glyoxalase I in Alzheimer's disease. Proc Natl Acad Sci U S A. 2004;101: 7687-7692

19. Politi P, Minoretti P, Falcone C, Martinelli V, Emanuele E. Association analysis of the functional Ala111Glu polymorphism of the glyoxalase I gene in panic disorder. Neurosci Lett. 2006;396: 163-166

20. Rehnstrom K, Ylisaukko-Oja T, Vanhala R, von WL, Peltonen L, Hovatta I. No association between common variants in glyoxalase 1 and autism spectrum disorders. Am J Med Genet B Neuropsychiatr Genet. 2008;147B: 124-127

21. Sacco R, Papaleo V, Hager J, Rousseau F, Moessner R, Militerni R, et al. Case-control and family-based association studies of candidate genes in autistic disorder and its endophenotypes: TPH2 and GLO1. BMC Med Genet. 2007;8: 11

22. Wu YY, Chien WH, Huang YS, Gau SS, Chen CH. Lack of evidence to support the glyoxalase 1 gene (GLO1) as a risk gene of autism in Han Chinese patients from Taiwan. Prog Neuropsychopharmacol Biol Psychiatry. 2008;32: 1740-1744

23. Sakhi AK, Berg JP, Berg TJ. Glyoxalase 1 enzyme activity in erythrocytes and Ala111Glu polymorphism in type 1-diabetes patients. Scand J Clin Lab Invest. 2013;73: 175-181

24. Abdul-Maksoud RS, Elsayed WS, Elsayed RS. The influence of glyoxalase 1 gene polymorphism on its expression at different stages of breast cancer in Egyptian women. Genes Cancer. 2017;8: 799-807

25. Peculis R, Konrade I, Skapare E, Fridmanis D, Nikitina-Zake L, Lejnieks A, et al. Identification of glyoxalase 1 polymorphisms associated with enzyme activity. Gene. 2013;515: 140-143
